# The impact of diabetes on labour market participation: a systematic review of results and methods

**DOI:** 10.1186/s12889-018-6324-6

**Published:** 2019-01-07

**Authors:** Sara Pedron, Karl Emmert-Fees, Michael Laxy, Lars Schwettmann

**Affiliations:** 1 Helmholtz Zentrum München (GmbH), Institute of Health Economics and Health Care Management, Ingolstädter Landstraße 1, 85764 Neuherberg, Germany; 2 German Center for Diabetes Research (DZD), Neuherberg, Germany

**Keywords:** Diabetes mellitus, Labour market, Indirect cost, Employment, Unemployment, Early retirement, Disability pension, Systematic review

## Abstract

**Background:**

Diabetes mellitus is a major chronic disease, which is connected to direct and indirect costs and productivity losses. However, its effects on labour market participation are not straightforward to identify, nor are they consistently included in cost-of-illness studies. First, this study aims to synthesise existing evidence regarding the impact of diabetes on labour market outcomes that imply a complete absence of work. Second, the analysis takes a particular look at relevant methodological choices and the resulting quality of the studies included.

**Methods:**

We conducted a systematic literature research (PubMed, Embase, PsychINFO), by applying a standard screening, selection and results extraction process, which considered all types of studies including cross-sectional and longitudinal approaches. Risk-of-bias and quality within the studies were assessed and results were compared. We dedicated special attention to the modelling of potential reverse causality between diabetes and labour market outcomes and the consideration of comorbidities and complications.

**Results:**

Overall, 30 studies satisfied our inclusion criteria. We identified four main labour participation outcomes: absence of employment, unemployment, early retirement, and disability pension. The studies reviewed show a negative impact of diabetes on the labour market participation outcomes considered. However, only a few studies controlled for endogeneity, differentiated between type 1 and type 2 diabetes or modelled the impact of comorbidities. We report how modelling choices affect the directions and interpretations of the effects.

**Conclusions:**

The available evidence mainly suggests a negative impact of diabetes on several outcomes indicating labour market participation. The methodological limitations identified can guide future research with respect to both outcomes and methods. This study provides therefore an empirical contribution to the discussion on how to model the economic impact of diabetes.

**Electronic supplementary material:**

The online version of this article (10.1186/s12889-018-6324-6) contains supplementary material, which is available to authorized users.

## Background

Diabetes mellitus is a major chronic disease with increasing public health relevance in high-, low- and middle-income countries. According to recent estimates, the number of people suffering from this condition worldwide will rise from 425 million in 2017 to 629 million by 2045 [1]. The progressing prevalence of this illness is especially due to type 2 diabetes, which constitutes 90–95% of diabetes cases, and the increasing average age of populations [2–5]. Due to this increase, total health care expenditures resulting from diabetes mellitus are estimated to rise from $727 billion in 2017 to $776 billion in 2045 [1].

Type 2 diabetes is closely linked to environmental and lifestyle risk factors, such as unhealthy diet, smoking and physical inactivity. Furthermore, the management of both type 1 and type 2 requires a high level of patient awareness and self-management [1]. For these reasons, many countries have established prevention and disease management programs to reduce incidence rates and to help affected people coping with the illness [6–8]. If poorly managed, both types of diabetes could lead to severe medical complications, which can affect an individual’s ability to work and may lead to lower productivity at work (presenteeism) or missing workdays (absenteeism) [9].

Existing systematic reviews suggest a clear effect of diabetes on economic costs [10, 11], work ability, work functioning, macroeconomic productivity and socio-economic consequences [9–11]. Despite this evidence, most cost-of-illness studies base their calculations of indirect costs on productivity losses due to short or long term morbidity (absenteeism, presenteeism and disability pension) and mortality [12]. However, as suggested by the American Diabetes Association [13], considering only these factors might result in a rather conservative approach, since individuals with diabetes might have lower workforce participation rates than the overall population, which would not be adequately captured simply accounting for such short and long term productivity losses. Although the underestimation caused by this flaw could be mitigated by adopting a friction cost approach, the effect remains of key importance in the correct computation of individual and general societal costs due to diabetes.

However, understanding and empirically estimating the effects of diabetes on workforce participation is not straightforward. The correct empirical strategy to examine the relationship between diabetes and workforce participation requires careful consideration of potential confounding, of reverse causality between the illness and workforce participation - otherwise termed “endogeneity” -, of different types of diabetes mellitus and of its associated complications.

Given the growing importance of diabetes, the complex assessment of its productivity losses, and the potential heterogeneity in the applied econometric methods to address this question, a careful pooling and critical assessment of the existing evidence regarding the impact of diabetes on labour market participation is needed. Therefore, the aim of the present review is twofold: First, we gather all existing evidence regarding the relation between diabetes and workforce participation outcomes (employment/unemployment, early retirement, and permanent disability pension). Second, we distinguish and assess methodological characteristics in existing studies. Hence, this review contributes to the discussion on the appropriate modelling of diabetes impact, provides methodological guidance for future studies and, therefore, fosters informed decisions in health policy and research.

## Methods

### Search strategy

The review was conducted and reported following the PRISMA guidelines [14]. We applied a structured approach, combining keywords and Medical Subject Headings (MeSH®) or Embase Subject Headings (Emtree®) on diabetes and labour market outcomes. The full set of the search terms for one database is represented in detail in Additional file 1. We applied the search on three databases: PubMed, Embase and PsychINFO. All databases were accessed using our institutional login. Additionally, at the end of the selection process, eligible studies, but also economic modelling studies focusing on the impact of diabetes on the selected outcomes, were screened for references.

### Inclusion and exclusion criteria

Included original studies had to be published in a peer-reviewed journal between 1st January 2000 and 28th March 2017 in any language and had to focus on the general population of adults aged 18–64. Papers focusing on women or specific ethnic groups were also considered eligible whereas studies, which only aim at specific subpopulations of patients suffering from other diseases were excluded. All articles screened by abstract had an English version of the abstract available, and for none of the eligible studies the use of a translator was necessary.

We focused on studies which evaluated the impact of diabetes or its biomarkers, such as hyperglycaemia or haemoglobin A1c (glycosylated haemoglobin) higher than 6.5% [15], on labour market outcomes indicating the complete absence of an occupation, i.e. employment, unemployment, early retirement or reception of a permanent disability pension, but not mortality or other measures of productivity covered in other reviews [9, 11]. In addition, studies were considered not eligible if diabetes appeared as a cluster of several conditions (e.g. metabolic disorders, cardiovascular risk factors) or if the outcome of interest could not be distinguished from other outcomes.

We included both cross-sectional and longitudinal studies with the primary aim of estimating the impact of diabetes on the selected outcomes, while economic modelling studies (cost-of-illness studies and simulations) were not included.

### Study selection process

After pooling the results in EndNote (Version X7) and eliminating duplicates, two authors (SP, KEF) carried out an independent three-step successive screening process of the articles regarding titles, abstracts and full-texts, by considering the predefined inclusion criteria described above. Disagreements were first discussed between the two authors and afterwards with the other authors (LS, ML).

### Data extraction and synthesis

After the identification of all eligible studies, we collected the results by using a predefined extraction form based on the well-established *Cochrane Consumers and Communication Review Group data template* [16]. From each paper we extracted a standardised set of information including the general characteristics of the study, the data source and the study population, the outcome measure considered and its definition, the analysis method used, the type of results reported, and finally the magnitude of corresponding effects. For those studies, which take endogeneity into account, we also added the necessary information allowing the evaluation of their methodological rigor. In all cases, missing information was retrieved by consulting survey web pages, reading explanatory publications or contacting authors. Furthermore, we grouped the studies in four different outcome categories. Studies which analysed the impact of diabetes on a binary variable indicating the presence of an occupation were grouped under the term “employment”. Other studies considered a binary variable indicating the absence of an occupation or the status “unemployment”, i.e. currently not employed but actively looking for an occupation. Furthermore, we identified two other clusters, i.e. studies which focused on “early retirement” and studies which focused on the full receipt of a permanent “disability pension”.

### Quality appraisal

We assessed the quality and the risk of bias of each eligible study based on the Newcastle-Ottawa Scale [17]. Two authors (SP and KEF) assessed each study independently and discussed disagreements with the other two authors. The scale entails three domains (selection, comparability, and exposure) with several sub-questions, focusing on representativeness of the dataset, measurement of exposure/outcome, and control variables included. Since the original scale is only available for cohort and case-control studies, we based our quality analysis on a modified version of the scale [9, 10]. Cross-sectional studies could be awarded a maximum of 6 points, while longitudinal studies had a maximum of 8 (see Additional file 2 for further details).

Due to the high heterogeneity of outcomes, we limited our analysis to a comparison of results based on their direction and level of significance. Furthermore, we focused our qualitative synthesis on methodological differences and how they influenced results in the studies. Finally, as a robustness check we focused our qualitative synthesis on studies which were awarded more than half of the maximum quality score indicating a low risk of bias.

## Results

### Description of included studies

**Fig. 1:**
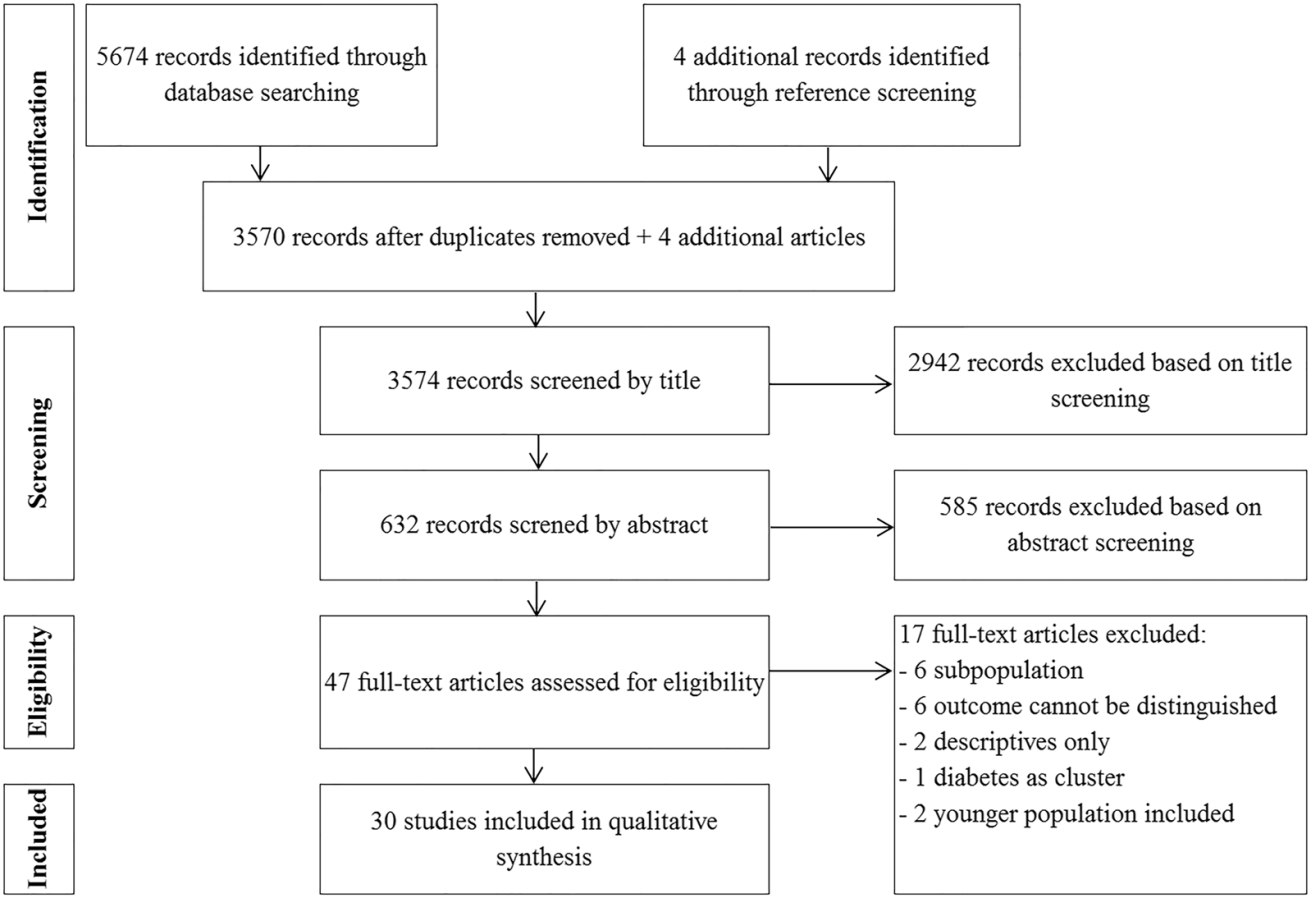
PRISMA flowchart

Our search yielded 5674 records, resulting in 3570 papers after elimination of duplicates (Fig. 1). Through reference screening we identified 4 other articles. After the three-step screening process, thirty studies were considered eligible for the qualitative synthesis (Fig. 1).

As reported in Table 1 and detailed in Table 2, nineteen out of these thirty studies had a cross-sectional design [18–36], ten had a longitudinal design [37–46] and one study used both kinds of designs [47]. Most studies were based on data from North America (15 studies), Europe (7 studies) or Australia (6 studies), while low and middle-income countries (LMICs) from Asia [24, 39] or Central America [31] were object of three studies only.

**Table 1:** Descriptive table of included studies

Category	Characteristics	Number of studies
Design^a^	Cross sectional	20
	Longitudinal	11
Context		
Area^a^	North America	15
	Europe	7
	Australia	6
	Asia	2
	Central America	1
Period of data collection	Before 2000	14
	After 2000	16
Dataset	Survey only	26
	Survey + Register	4
Participants		
Number of participants	< 10,000	11
	≥10,000 to < 50,000	13
	≥50,000 to < 100,000	5
	> 100,000	1
Population	General population	28
	Employees in the energy sector	1
	Employees in the public sector	1
Sex	Both	27
	Only Women	3
	Only Men	0
Age^b^	18 or older	16
	45 or older	7
	50 or older	7
Definitions		
Diabetes definition	self-report	25
	register data	3
	laboratory analysis	2
Diabetes type^a^	T1DM only^c^	1
	T2DM only^c^	1
	Both distinguished	4
	Both undistinguished	24
	Haemoglobin A1c > 6.5%	1
Outcome^a^	Employment	16
	Unemployment	8
	Early retirement	8
	Disability pension	5

^a^These studies do not sum up to 30. Some studies included more than one of the characteristics indicated

^b^The indicated age refers to the youngest participant. Generally, the studies included only people maximum 64 or 65 years old. For details see Table 2

^c^T1DM: type 1 diabetes mellitus; T2DM: type 2 diabetes mellitus

**Table 2:** Eligible studies evaluating the effect of diabetes on labour market outcomes

Study	Methods					Results							Other			
	Study design^a^	Outcome definition	Age group	Exposure	Statistical method	Summary measure^d^	Overall		Men		Women		Confounders^e^	Comorbidities/complications modelling	Endogeneity^g^	Quality score
Employment																
Ng et al. (2001) [27]	C	Currently working (vs. currently not working)^b^	18–65	Diabetes	Probit regression	PC	−0.04	*					A, CC, E, F, G, L, MS, SH	Stratification	no	5/6
				T1DM	Probit regression	PC	0.11	*								
Bastida et al. (2002) [19]	C	Currently working (vs. currently not working)	45+	Diabetes	Probit regression	ME			− 0.08	*	− 0.07		A, E, F, H, I, MS, O	–	no	5/6
Yassin et al. (2002) [34]	C	Being employed for most of the time in the last 12 months	18–64	Diabetes	Multinomial logistic regression	OR			0.53		0.48	*	A, E, I, MC, MS, O, SM	–	no	5/6
Brown et al. (2005) [20]	C	Currently working (vs. currently not working)^b^	45+	Diabetes	Probit regression	PC			−1.02	*	− 0.34	*	A, E, F, H, I, MS, O	–	yes	5/6
					Recursive bivariate probit IV	PC			−1.71	*	0.51					
Klarenbach et al. (2006) [22]	C	Working at a job or business and being present at that job for the week before	20–59	T2DM	Logistic Regression	OR	0.70	*					A, CC, E, G, L, MS, O	Confounders	no	4/6
Harris (2009) [21]	C	Currently employed (vs. not working but not retired)	> 25	Diabetes	Endogenous multivariate probit model	ME			−0.07	*	− 0.09	*	A, CC, E, F, I, MS, PA, SM	Confounders	yes	4/6
Latif (2009) [23]	C	Having had a job in the last 12 months	15–64	Diabetes	Probit regression	PC			−0.65	*	− 0.44	*	A, E, H, L, MS	–	yes	5/6
				Diabetes	Recursive bivariate probit regression IV	PC			0.96		0.19					
Zhang et al. (2009) [36]	C	Currently working (vs. currently not working)^b^	18–49	Diabetes	Endogenous recursive multivariate probit model	TE (%)			−3.91	*	− 3.70		A, CC, E, MS, O, Y	Confounders	yes	4/6
			50–64	Diabetes	Endogenous recursive multivariate probit model	TE (%)			−11.47	*	− 0.20					
Lin (2011) [24]	C	Currently working (vs. currently not working)	45–65	Diabetes	Recursive bivariate probit model	ME	−0.24	*	− 0.19	*	− 0.15		A, E, G, I, MS	–	yes	5/6
Minor (2011) [25]	C	Worked for pay at some point during the last year	20–65	Diabetes	IV estimation (model 1)	ME	−0.42	*					A, E, F, F, J, L, MS, O, SH	–	yes	5/6
				T1DM	IV estimation (model 2)	ME	−0.06									
				T2DM		ME	−0.45	*								
Seuring et al. (2015) [31]	C	Having worked or carried out an activity that helped with the household expenses for at least 10 h over the last week	15–44	Diabetes	Probit regression	ME			− 0.01		0.00		A, E, F, I, L, MS, O, PE	–	yes	5/6
			45–64			ME			−0.110	*	− 0.06	*				
Nielsen et al. (2016) [28]	C	Currently working (vs. currently not working)	18–103	T1DM	Linear regression	RD	−9.10	*	− 5.30	*	− 12.20	*	A, E, G, SH	–	no	4/6
Minor et al. (2016) [26]	C	Currently working (vs. currently not working)	18–65	A1c levels > 6.5%	Probit regression (model 1)	ME			−0.02		− 0.16		A, E, F, MS, O, Y	–	no	5/6
				T2DM		ME			−0.11	*	− 0.19	*				
				T1DM		ME			−0.17		0.18	*				
				T2DM	Probit regression (model 2)	ME			−0.09	*	− 0.19	*				
				T1DM		ME			−0.16		0.175	*				
Tunceli et al. (2005) [46]	L	Working for pay outside the home (vs. Not working for pay outside home)	51–61	Diabetes	Probit regression (model 1)	ME			−0.09	*	− 0.06	*	A, BMI, E, F, I, J, MS, O	Confounders SA^f^	no	6/8
				Diabetes	Probit regression (model 2)	ME			−0.07	*	− 0.04		A, BMI, E, F, I, J, MS, O, CC			
Pit et al. (2012) [44]	L	Employment last week (more than one hour spent on an occupation with or without pay) (vs. less than one hour spent last week on an occupation or unemployed)	51–61	Diabetes	Robust nested multivariate longitudinal analyses (GEE)	OR	0.82	*					BMI, CC, E, F, L, MS, SM, Y	Confounders	no	4/8
Minor (2013) [43]	L	Currently working (vs. currently not working)^b^	45–53	T1DM	Logistic regression	LC			0.22		−0.03		A, E, F, FH, J, L, MS, O, Y	Confounders SA^f^, modelling time from diagnosis	no	6/8
				T2DM		LC			−0.42	*	− 0.37	*				
				T1DM	Logistic regression	LC			0.02		0.28		A, BMI, CC, E, F, FH, J, L, MS, O, Y			
				T2DM		LC			−0.28		−0.36	*				
Unemployment																
Alavinia et al. (2008) [18]	C	Currently unemployed (vs. Having done any kind of paid work in the last four weeks)	50–65	Diabetes	Logistic regression	OR	1.38						A, AL, BMI, CC, E, G, MS, PA, SM	Confounders	no	4/6
Smith et al. (2014) [32]	C	Currently not employed due to health reasons (vs. currently employed)	25–74	Diabetes	Logistic regression	OR	2.22	*					A, BMI, CC, E, F, G, I, L, MS, Y	Confounders	no	3/6
Van Der Zee-Neuen et al. (2017) [33]	C	Currently unemployed (vs. currently employed)	18–65	Diabetes	Multinomial logistic regression	OR	1.88						A, BMI, E, G, SM	–	no	4/6
Yassin et al. (2002) [34]	C	Transition from employment to no employment due to health reasons	18–64	Diabetes	Logistic regression	OR			3.1	*	2.9		A, E, I, MC, MS, O, SM	–	no	5/6
Rumball-Smith et al. (2014) [30]	C	More than one year of absence from the labour force or retirement (vs. Currently employed)	> 50	Diabetes	Cox proportional hazards models (matching diabetes subject with seven non-diabetes matches)	HR	1.30	*	1.26	*	1.34	*	A, E, G, L	–	no	5/6
Kraut et al. (2001) [41]	L	Not in the labour force (not employed and not seeking job) vs. in the labour force	18–64	Diabetes (w comp)	Logistic regression	OR	2.07	*					A, G, L, MS, O	Exposure	no	6/8
				Diabetes (w/o comp)	Logistic regression	OR	1.20									
		Unemployed (no job but actively looking for it) vs. employed (with job)	18–64	Diabetes	Logistic regression	OR	1.45									
				Diabetes (w comp)	Logistic regression	OR	1.69									
				Diabetes (w/o comp)	Logistic regression	OR	1.35									
Kouwenhoven-Pasmooij et al. (2016) [40]	L	Transition from employment to unemployment	> 50	Diabetes	Multinomial logistic regression	OR	1.17						A, CC, E, G, L, MS,	Confounders	no	6/8
Majeed et al. (2015) [42]	L	“Early paid work” (vs. “mostly in the labour force”)^c^	45–50	Diabetes	Multinomial logistic regression	OR	1.44	*					BMI, E, F, I, MS, SM	–	no	4/8
Early retirement																
Vijan et al. (2004) [47]	C	Currently retired (vs. currently working)	51–61	Diabetes	Logistic regression	OR	1.3						A, E, F, G, MS, O	–	no	4/6
Alavinia et al. (2008) [18]	C	Currently retired (vs. Having done any kind of paid work in the last four weeks)	50–65	Diabetes	Logistic regression	OR	1.33	*					A, AL, BMI, CC, E, G, MS, PA, SM	Confounders	no	4/6
Pit et al. (2013) [29]	C	Retirement due to health reasons (vs. Working)	45–65	Diabetes	Multinomial logistic regression	OR			1.44	*	1.30		A, CC, E, MS	Confounders	no	3/6
		Retirement for other reasons (vs. Working)							1.16		1.07					
Yen et al. (2011) [35]	C	Age at retirement	50–75	Diabetes at age 50	OLS regression	OLS	−1.39	*					CC, E, G, I, J, L, O	Confounders	no	3/6
Vijan et al. (2004) [47]	L	Incremental duration of retirement over the 8 years follow-up	51–61	Diabetes at baseline	Two-part multivariable model (logistic regression + OLS)	OLS	0.14	*					A, E, F, G, MS, O	–	no	6/8
Shultz et al. (2007) [45]	L	Transition from employment to retirement	47–64	Diabetes at baseline	Multinomial logistic regression	OR	3.37	*					A, CC, G, I, O	Confounders	no	4/8
Herquelot et al. (2011) [38]	L	Transition from employment to retirement	35–60	Diabetes (in at least three consecutive yearly questionnaire)	Cox proportional-hazard regression	HR	1.6	*					A, BMI, G, J	–	no	6/8
Kang et al. (2015) [39]	L	Transition from employment to early retirement due to health problems	45–70	Diabetes at baseline	Cox proportional hazard model	HR	1.47	*	1.52		1.40		A, AL, BMI, CC, G, I, J, PA, SH, SM	Confounders	no	5/8
Kouwenhoven-Pasmooij et al. (2016) [40]	L	Transition from employment to retirement	> 50	Diabetes	Multinomial logistic regression	OR	1.06						A, CC, E, G, L, MS,	Confounders	no	6/8
Disability pension																
Vijan et al. (2004) [47]	C	Currently receiving a disability pension (vs. currently working)^a^	51–61	Diabetes	Logistic regression	OR	3.1	*					A, E, F, G, MS, O	–	no	4/6
Van Der Zee-Neuen et al. (2017) [33]	C	Currently receiving a disability pension (vs. Currently employed)	18–65	Diabetes	Multinomial logistic regression	OR	2.32	*					A, BMI, E, G, SM	–	no	3/6
Vijan et al. (2004) [47]	L	Incremental duration of disability pension over the 8 years follow-up	51–61	Diabetes at baseline	Two-part multivariable model (logistic regression + OLS estimation)	Cumulative impact of diabetes (years)	0.79	*					A, E, F, G, MS, O	–	no	6/8
Herquelot et al. (2011) [38]	L	Transition from employment to disability pension	35–60	Diabetes (in at least three consecutive years)	Cox proportional-hazard regression	HR	1.4						A, BMI, G, J	–	no	6/8
Ervasti et al. (2016) [37]	L	Transition from employment to disability pension	30–65	Diabetes at baseline (vs. No metabolic condition)	Cox proportional-hazard regression (model 1)	HR	1.84	*					A, G, SES	Confounders SA^f^	no	7/8
				Diabetes at baseline (vs. No metabolic condition)	Cox proportional-hazard regression (model 2)	HR	1.56	*					A, AL, BMI, CC, G, J, PA, SES, SM			
Kouwenhoven-Pasmooij et al. (2016) [40]	L	Transition from employment to disability pension	> 50	Diabetes or high blood glucose levels	Multinomial logistic regression	OR	2.37	*					A, CC, E, G, L, MS,	Confounders	no	6/8

*p-value< 0.05

^a^C: cross-sectional study; L: longitudinal study;

^b^Not clearly stated but understood from context, interpretation, questions asked in survey

^c^Other outcomes considered (“increasingly paid work”, “gradually not in paid work”, “mostly not in paid work”) are not reported here

^d^OR: Odds Ratio HR: Hazard Ratio ME: Marginal Effect PC: Probit Coefficient LC: Logit Coefficient TE: Treatment Effect RD: Risk Differences OLS: OLS-Coefficient

^e^*A* Age, *AL* Alcohol use, *BMI* Body-Mass-Index, *CC* Comorbidities/complications, *E* Education/Years of schooling, *F* Family related features (Number of children; Family size; People living in houehold; Household size; Living with someone who needs care; Competing activities;); *FH* Family health, *G* Gender, *H* Owns home, *I* Income/Wealth, *J* Employment characteristics, (Self-employment; Job tenure; Work experience; Part time; Occupational status;) *L* Region, Area of living/residence, *MC* Medical cost, *MS* Marital status, *O* Origin (Race, Australian born, Immigrant status) 0 *PA* Physical activity, *PE* Parental education, *SH* Subjective health/health related quality of life, *SM* Tobacco use/Smoking, *Y* Year

^f^Complications were used in the sensitivity analysis as confounders;

^g^Presence of endogeneity: yes = endogeneity of diabetes was detected; no = endogeneity of diabetes was not detected

Other information (e.g. sample size, country, method of data collection, results of IV tests) are not included in the table due to space limitations and are available from the corresponding author upon request

Most data were collected through large population-based surveys, while only four of these studies linked those data to morbidity or administrative registries [28, 37, 38, 40]. Almost half of the studies evaluated recent data collected from the year 2000 onwards. The other half analysed data collected in the last century, dating back until 1979.

Only a minority of studies focused on specific groups of employees [37, 38] or women [25, 42, 44], whereas the majority considered (population-based) samples from the general population.

While half of the studies focused on the elderly, the other half included samples from the whole working age population (aged 18–64). However, they generally carried out a stratified analysis for different age groups, so that the results are generally comparable among all studies on this regard.

Table 2 and Additional file 2 show that no study reached the maximum quality score. Three cross-sectional and three longitudinal studies gained half of the available points. This indicates a high risk of bias. The majority of studies were assigned a low score because, among other reasons, they used self-reported diabetes status as the exposure variable. Only a few studies based their analysis on more objective information from morbidity registries or formal blood tests [21, 26, 28, 37, 41]. Furthermore, the studies which adopted an “objective” definition of diabetes did not clearly indicate which pieces of information were used to carry out such definition, i.e. whether the status was defined on the basis of blood parameters (glycosylated haemoglobin, fasting or plasma glucose) or on the basis of a previous medical diagnosis reported by the participants. Additionally, in most cases the labour market outcomes were defined through structured interviews or questionnaires, resulting in a low scoring for several studies (Additional file 2).

In Table 2, we clustered the available studies according to the outcome(s) of interest. Despite the sorting into similar outcomes groups, the definitions of outcomes and control groups still varied considerably within each cluster, limiting the comparability of the studies included. For these reasons, any generalized comment or comparison of effect magnitude among the studies in this framework is not feasible, unfortunately.

### Effects on employment

In general, as can be inferred from Table 2, the studies show a negative and statistically significant association between the presence of diabetes and the outcome “employment”. However, the magnitude of the effect varies greatly between the studies considered. This might be due to differences in the mean sample ages, modelling techniques or outcome definitions. This negative effect does not change when we focus only on studies with a low risk of bias. Furthermore, considering the overall evidence, statistically significant coefficients for both genders are reported. However, within studies, estimated coefficients are generally higher in men than in women, indicating a stronger effect of diabetes on employment in males (see Table 2).

When focusing on studies from LMICs [24, 31, 39], diabetes does not show any effect on the employment chances of women, while the effect for men remains negative. This finding is in line with the overall results, but shows a much lower, if non-existent, effect in women compared to the other studies from HIC (high income countries). Furthermore, a few studies differentiated the analysis between type 1 and type 2 diabetes (T1DM and T2DM) [25–28, 43]. They show that the negative effect of diabetes is actually driven by T2DM, since the coefficients on type 1 are generally insignificant or in some cases positive and statistically significant.

By applying different statistical methods, seven out of thirteen cross-sectional studies considering employment as the outcome variable tested for endogeneity of diabetes. In order to take this factor into account, authors employed either recursive multivariate probit models, jointly estimating the probability of diabetes, other comorbidities/complications (cardiovascular disease, depression, etc.) and employment, or used an instrumental variable (IV) approach, when genetic information (diabetes status of parents or siblings) was available. Not all studies detected the presence of endogeneity. Furthermore, if endogeneity was found to be present, modelling approaches aiming to account for endogeneity revealed either an under- or an overestimation of the coefficients compared to models without endogeneity. Hence, the overall picture is rather inconsistent.

To model the presence of comorbidities or complications, some studies included relevant variables as confounders in the analysis, without discussing the implications of their modelling choices [21, 22, 36, 44]. In contrast, other authors used these factors as additional controls in more complex model specifications, discussing their role with regard to the magnitude and the significance of the coefficients compared with simpler model specifications [43, 46]. As a result, some coefficients on the diabetes variable lost their significance (see Table 2) or decreased (c.f. Table 2). In contrast, Ng et al. (2001) [27] carried out an additional analysis focusing only on the diabetes group and tested the impact of comorbidities. Their analysis shows that people suffering from diabetes and other comorbidities have a 12% lower probability of being in the labour force than people suffering from diabetes but without any complication.

### Effects on unemployment

For the second outcome considered, i.e. “unemployment”, heterogeneity in the outcome definition is particularly apparent. Groups of employed individuals are compared with very different samples of persons without occupation. From pooling corresponding evidence, it emerges that the presence of diabetes has no impact on the probability of having no occupation but still being economically active [18, 33, 40, 41]. However, it is associated with a complete exit from the labour market [30, 32, 34, 41]. Furthermore, by differentiating the exposure variable in diabetes with/without complications, Kraut et al. (2001) [41] revealed that people suffering from diabetes with complications are more likely to exit the labour force compared to individuals not suffering from diabetes, whereas this observation does not hold for people with diabetes without complications.

### Effects on early retirement and permanent disability pension

In general, studies focusing on early retirement revealed a positive association between the presence of diabetes and the probability of retiring early. In contrast, two studies stratified their analyses with respect to gender and revealed only weak evidence for either women or men [29, 39]. However, one of these studies shows a high risk of bias [29], while the other one entails a low number of observations, probably leading to a lack of significance in the final result [39].

Studies evaluating the fourth outcome, viz. “permanent disability pensions”, revealed a positive association with the presence of diabetes. In the paper by Ervasti et al. (2016) [37] several models with different comorbidities and complications are reported. After introducing corresponding confounders, coefficients on diabetes remained positive and statistically significant, but their magnitude diminished (see Table 2).

### Robustness checks

Generally, leaving out studies at high risk of bias does not change the pattern of synthesised results remarkably for different outcomes. No study focusing on unemployment, early retirement or disability pension distinguished between T1DM and T2DM or considered endogeneity of diabetes. Furthermore, only two studies stratified the analysis for gender, and several studies included comorbidities or complications as confounders, potentially adding other sources of bias to the analysis.

## Discussion

### Summary of evidence and interpretation

We identified 30 studies, which evaluated the impact of diabetes on labour market outcomes, which imply a complete absence of any occupation. The available studies were quite heterogeneous in terms of definition of outcomes, age of the population considered and statistical method used even within the four outcome clusters we identified. Generally, the studies included provide consistent evidence that diabetes is negatively associated with employment and that diabetes patients are more likely to retire early, be fully out of the labour force and to receive a full and permanent disability pension, although effects may vary across subgroups.

The studies included also show considerable differences in the methods used, which could significantly impact the results. Furthermore, evaluations are often based on an extremely simplified modelling of diabetes, its dynamics and its progression, resulting in potential sources of bias. In this context, the majority of data is based on self-reported diabetes status and often no heterogeneity factors or endogeneity of the labour market outcomes are considered, resulting in lower quality scores for several studies included.

Specifically, a stratified analysis using potential sources of heterogeneous effects, such as gender, age, age at retirement or diabetes type, was inconsistently carried out throughout the studies, limiting the comparison of results regarding different groups within the scope of this review. In fact, a consistent stratified analysis between genders is available only for the outcome “employment”. For the other outcomes, only isolated evidence with a high risk of bias could be found [29, 30, 34, 39]. As shown in many of the studies included [16, 19, 25, 27, 30, 32, 35, 39, 42] and in a previous review [7], both men and women suffering from diabetes have higher chances of adverse labour market outcomes, but within the same studies, the effect is generally higher for men than for women. However, no study furnished an evidence-based explanation of this result. The main interpretation is that, since the employment chances of elder females are already low due to several other factors (e.g. providing informal care, traditional household regimes), diabetes influences the employment chances of women in a less disruptive way than those of men. In this context, also the differences between studies from LMICs and other countries should be emphasized: the effect of diabetes for the employment and early retirement chances of women in LMICs is never significant, while the effect for men is in line with those observed in HIC [24, 31, 39]. The non-significant effect for women should be put in the right context and should be interpreted in the light of labour market differences, regarding most notably the social security systems and the role of women in society, which still characterize the divide between HIC and LMICs and which could significantly affect the employment chances of women in the first place. However, in line with previous studies [11], this review highlights also the paucity of evidence regarding the differences between HIC and LMICs, since only three of the included studies focused on the latter [24, 31, 39], and thus highlights the need for more research on these differences.

Most studies were based on large survey data, where diabetes status was self-reported (see Table 1). Although previous studies showed that there is a high correspondence between self-report and objective diagnosis [48, 49], this implies that most of the available evidence regarding the effect of diabetes on labour market outcomes bases its analysis and conclusions on a subjective measure of diabetes and is thus potentially open to bias. This bias is expected to be upwards, since the undiagnosed cases are probably those who also do not show any symptom or impairment from the disease, and as such are much less likely to leave the labour force due to diabetes. This potential pitfall is reflected in the lower quality score assigned to those studies based on self-report of diabetes and should be considered as an important limitation of the available evidence in this field.

Furthermore, in the same studies, no other information about age at onset, diabetes type, severity or medications was available, according to the publications identified. One important distinction in this context is that between T1DM and T2DM. Although the prevalence of T1DM is usually low [1], not controlling for this difference could cause a downward bias and, thus, an underestimation of the effect of T2DM on employment. In fact, the few studies that distinguish between the two diabetes types show that the negative effect of diabetes on employment is actually driven by T2DM, since the coefficients on T1DM are either insignificant or even significantly positive. Furthermore, T1DM and T2DM are two distinct conditions, with two different aetiologies and ways of coping with the illness. Therefore, this difference should be taken into account when modelling diabetes. For example, in absence of more detailed information, the age at onset could offer a good approximation, as already done in some of the studies included [25, 26].

Most studies also adopted a very simplified modelling of comorbidities and complications. These factors can play a crucial role in the ability to work of diabetes patients over the life course and, thus, should be considered when modelling diabetes and labour market outcomes. There is no consensus on how to take them into account. In most of the studies considered, they are either not taken into account or are modelled as confounders. However, as highlighted by some authors [25], simply adding them as confounders could be problematic, since they might be highly correlated with diabetes or a result of common unobserved factors. Therefore, including them as covariates into the model could result in biased estimates for the diabetes variable. In isolated cases comorbidities and complications are included [1] as confounders in different versions of the model as further specification [37, 43, 46], [2] as a way to differentiate the exposure variable (diabetes with/without complications) [41] or [3] as exposure in a further analysis focusing only on the diabetes group [27]. These three implementations show that adding such confounders leads to a change in the magnitude or in the significance of the coefficient on the diabetes variable [37, 43, 46]. In addition, Kraut et al. (2001) [41] showed that only diabetes with complications leads to a full labour market exit. Ng et al. (2001) [27] also revealed that people suffering from diabetes with complications have a higher chance of being out of the labour force than people suffering from diabetes without complications.

A further issue, only addressed in a few studies, is the problem of reverse causality or endogeneity of diabetes in labour market outcome models. Typical ways for taking this problem into account include recursive multivariate probit approaches [20, 21, 23, 24] or the use of genetic instrumental variables [25, 31]. Results from studies taking endogeneity into account generally differed in two aspects: (i) the actual endogeneity of the diabetes variable and (ii) the direction of the bias in the regression coefficients with respect to the basic model without endogeneity. Overall, diabetes was not found to be consistently endogenous in each study considered and for every gender subgroup. Furthermore, while comparing the results from models with and without endogeneity within the same study, no clear direction of the bias of the coefficients could be highlighted (see Table 2). Therefore, since the pattern of presence and effect is not clear, endogeneity should always be tested for in this context and the limitations of results should be discussed carefully.

### Strengths and limitations

This review specifically gathered evidence regarding the effect of diabetes on all labour market outcomes involving the complete absence of occupation. Hence, it complements related reviews, which focused on other productivity outcomes [9] or reviewed part of the included outcomes as a secondary aim [11]. Furthermore, in the present review, we paid specific attention to the methods used, providing ground for an evidence-based discussion on how to produce credible and robust findings both from an economic and a statistical point of view.

However, our study may suffer from some limitations. First, we have adopted rather restrictive inclusion criteria. We searched three databases and we included only articles already published in peer-reviewed journals, starting from the year 2000. Therefore, the review might suffer from publication bias. However, the large number of studies initially retrieved after an independent screening by two researchers and a comprehensive reference check allowed us to apply such restrictive criteria in order to report the most robust evidence available. Second, we based our quality and risk of bias assessment on the Newcastle-Ottawa Scale [17], as already done in similar reviews [9, 10]. Besides the transparent procedure of evaluation, the scale had to be modified for our specific case, which prevents comparability to a certain extent (for detailed explanation see Additional file 2). Furthermore, the scale is actually suitable for evaluating epidemiological studies involving clinical outcomes but could still be adapted to our specific question and context. Although the scale represents the best instrument available to our knowledge, this problem should be taken into account in further studies, aiming at improving also quality and risk of bias assessment.

### Implications for practice, policy, and research

The aggregated evidence available reveals that generally, individuals suffering from type 2 diabetes mellitus are more likely to fully exit the labour market early, retire early and receive a permanent disability pension. Both men and women are affected, but the probability of employment of men is affected stronger than that of women. Diabetes can be endogenous in the labour market outcomes, but it is not clear why and in which cases it is present and how coefficients are influenced.

Maintaining and possibly also extending the ability to work of older workers is one of the primary goals of current pension reforms. This study shows, however, that chronically ill individuals suffering from T2DM, might not be able to maintain their employment status and will therefore exit the labour market earlier. Since T2DM prevalence is rising, not only in high- but also in low- and middle-income countries [1], a considerable effort should be undertaken to improve and prolong the ability to work of diabetes individuals. Specific attention should be paid to developing and increasing the efficacy of evidence-based prevention and management programs.

Finally, the existing evidence should be improved, specifically investigating the underlying dynamics and establishing and strengthening the link to practice. First, future cost studies investigating the indirect costs of diabetes should take the complete absence of an occupation due to diabetes or its complications into account. Failing to consider this aspect could lead to a severe underestimation of the burden this condition is imposing. Second, future studies will need to differentiate between gender and/or diabetes type, while also checking specifically for the endogeneity of diabetes. These methods should be applied for every outcome, not only for the presence versus absence of employment. Third, the issue of diabetes endogeneity should be discussed for each study, since no pattern of presence and effect could be found. Understanding how the underlying processes and effects work, being it through reverse causality or through unobserved factors, could also prove helpful in understanding how a chronic life-style illness impacts the outcomes considered. Lastly, the available studies adopt an extremely simplified definition and modelling of diabetes, its progression, its severity and its complications and comorbidities. Further research should rely on more objective ways to determine diabetes. Also, it should improve the understanding of which factors and dynamics actually lead to adverse labour market outcomes and should include different modelling strategies on how comorbidities and complications actually work. Furthermore, additional aspects of the illness, such as efficiency of management, health literacy, and medication adherence [50, 51], should be included in the analysis, to gather further understanding on underlying factors and allow for the individualisation of concrete starting points for practical intervention.

## Conclusions

This systematic literature review indicates that type 2 diabetes mellitus, but not type 1, is associated with lower productivity. We further found that the effect of diabetes is generally stronger in men than in women. In addition, the present study reveals that one of the largest potential sources of bias is the use of self-reported measures of diabetes, not confirmed by physicians or formal blood tests. Finally, the studies showed no consensus regarding the correct modelling strategy of diabetes and labour market outcomes. Only some of them considered possible endogeneity, or only partly discussed their modelling choices regarding the role of complications and comorbidities. Thus, the review highlights the need for improving the current practice of modelling diabetes and for understanding how the illness is connected with the outcomes. This is not only important for the accurate determination of indirect costs, but could also prove useful in the establishment of evidence-based prevention and disease management programs.

## Additional files

Additional file 1: Search strategy. This file contains a detailed account of the databases and terms/keywords and restrictions used in our search strategy for one database (PubMed). (PDF 156 kb)

Additional file 2: Newcastle-Ottawa Scale and quality scores. This file contains a detailed overview and explanation of the Newcastle-Ottawa Scale used to assess quality of the retrieved studies. The file entails also a detailed overview of the scores for each study in each dimension. (PDF 271 kb)

## Abbreviations

T1DM: Type 1 Diabetes Mellitus
T2DM: Type 2 Diabetes Mellitus
Haemoglobin A1c: glycosylated haemoglobin
LMIC: Low and middle income countries
HIC: High income countries
