# Supplementary material for: The impact of diabetes on labour market participation: a systematic review of results and methods
Source: BMC Public Health. 2019 Jan 7;19:25. doi: 10.1186/s12889-018-6324-6 (PMC6323654; doi:10.1186/s12889-018-6324-6)
Supplement: Supplementary file 1 — Search strategy. This file contains a detailed account of the databases and terms/keywords and restrictions used in our search strategy for one database (PubMed). (PDF 156 kb) [file 12889_2018_6324_MOESM1_ESM.pdf]

The impact of diabetes on labor market participation: a systematic review of results and methods

Sara Pedron, Karl Emmert-Fees, Michael Laxy, Lars Schwettmann*

BMC Public Health

Additional file 1: Search strategy

*corresponding author

Additional file 1: Search strategy

Database: PubMed

Complete search:

("Diabetes Mellitus"[Mesh]
OR "Hypoglycemia"[Mesh]
OR "Hyperglycemia"[Mesh]
OR "Hemoglobin A, Glycosylated"[Mesh]
OR diabetes[TIAB]
OR hypoglycemia[TIAB]
OR hyperglycemia[TIAB]
OR "glycosylated hemoglobin"[TIAB]
OR "HbA1c"[TIAB])
AND
(Efficiency[MeSh]
OR Retirement[MeSh]
OR Employment[MeSh]
OR "productivity"[TIAB]
OR "efficiency"[TIAB]
OR "lost productivity"[TIAB]
OR "lost productive time"[TIAB]
OR "lost time"[TIAB]
OR "retirement"[TIAB]
OR "employment"[TIAB]
OR "employment status"[TIAB]
OR "unemployment"[TIAB]
OR "employment outcome"[TIAB]
OR "vocational outcome"[TIAB]
OR "getting fired"[TIAB]
OR "burden of diabetes"[TIAB]
OR "work loss"[TIAB]
OR "work productivity"[TIAB]
OR "work disability"[TIAB]
OR "work participation"[TIAB]
OR "work cessation"[TIAB]
OR "work status"[TIAB]
OR "job loss"[TIAB]
OR "job productivity"[TIAB]
OR "job disability"[TIAB]
OR "job participation"[TIAB]
OR "job cessation"[TIAB]
OR "job status"[TIAB]
OR "workers loss"[TIAB]
OR "workers productivity"[TIAB]
OR "workers disability"[TIAB]
OR "workers participation"[TIAB]
OR "workers cessation"[TIAB]
OR "workers status"[TIAB]
OR "worker loss"[TIAB]
OR "worker productivity"[TIAB]
OR "worker disability"[TIAB]
OR "worker participation"[TIAB]
OR "worker cessation"[TIAB]
OR "worker status"[TIAB]
OR "workforce loss"[TIAB]
OR "workforce productivity"[TIAB]
OR "workforce disability"[TIAB]
OR "workforce participation"[TIAB]
OR "workforce cessation"[TIAB]
OR "workforce status"[TIAB]
OR "vocation loss"[TIAB]
OR "vocation productivity"[TIAB]
OR "vocation disability"[TIAB]
OR "vocation participation"[TIAB]
OR "vocation cessation"[TIAB]
OR "vocation status"[TIAB]
OR "vocational loss"[TIAB]
OR "vocational productivity"[TIAB]
OR "vocational disability"[TIAB]
OR "vocational participation"[TIAB]
OR "vocational cessation"[TIAB]
OR "vocational status"[TIAB]
OR "occupation loss"[TIAB]
OR "occupation productivity"[TIAB]
OR "occupation disability"[TIAB]
OR "occupation participation"[TIAB]
OR "occupation cessation"[TIAB]
OR "occupation status"[TIAB]
OR "occupational loss"[TIAB]
OR "occupational productivity"[TIAB]
OR "occupational disability"[TIAB]
OR "occupational participation"[TIAB]
OR "occupational cessation"[TIAB]
OR "occupational status"[TIAB]
OR "employment loss"[TIAB]
OR "employment productivity"[TIAB]
OR "employment disability"[TIAB]
OR "employment participation"[TIAB]
OR "employment cessation"[TIAB]
OR "employment status"[TIAB]
OR "labour force loss"[TIAB]
OR "labour force productivity"[TIAB]
OR "labour force disability"[TIAB]
OR "labour force participation"[TIAB]
OR "labour force cessation"[TIAB]
OR "labour force status"[TIAB]

OR "labor force loss"[TIAB]
OR "labor force productivity"[TIAB]
OR "labor force disability"[TIAB]
OR "labor force participation"[TIAB]
OR "labor force cessation"[TIAB]
OR "labor force status"[TIAB]
OR "workforce loss"[TIAB]
OR "workforce productivity"[TIAB]
OR "workforce disability"[TIAB]
OR "workforce participation"[TIAB]
OR "workforce cessation"[TIAB]
OR "workforce status"[TIAB]
OR "work-force loss"[TIAB]
OR "work-force productivity"[TIAB]
OR "work-force disability"[TIAB]
OR "work-force participation"[TIAB]
OR "work-force cessation"[TIAB]
OR "work-force status"[TIAB]
OR "employee loss"[TIAB]
OR "employee productivity"[TIAB]
OR "employee disability"[TIAB]
OR "employee participation"[TIAB]
OR "employee cessation"[TIAB]
OR "employee status"[TIAB]
OR "employment loss"[TIAB]
OR "employment productivity"[TIAB]
OR "employment disability"[TIAB]
OR "employment participation"[TIAB]
OR "employment cessation"[TIAB]
OR "employment status"[TIAB]
OR "leaving work"[TIAB]
OR "leaving job"[TIAB]
OR "leaving occupation"[TIAB]
OR "leaving employment"[TIAB]
OR "leaving labour force"[TIAB]
OR "leaving labor force"[TIAB]
OR "leaving workforce"[TIAB]
OR "leaving work-force"[TIAB]
OR "work incapacity"[TIAB]
OR "work incapability"[TIAB]
OR "worker incapacity"[TIAB]
OR "worker incapability"[TIAB]
OR "workers incapacity"[TIAB]
OR "workers incapability"[TIAB]
OR "working incapacity"[TIAB]
OR "working incapability"[TIAB]
OR "workforce incapability"[TIAB]
OR "workforce incapability"[TIAB]
OR "work-force incapability"[TIAB]
OR "work-force incapability"[TIAB]
OR "vocational incapacity"[TIAB]
OR "vocational incapability"[TIAB]
OR "occupational incapacity"[TIAB]
OR "occupational incapability"[TIAB]
OR "employment incapacity"[TIAB]
OR "employment incapability"[TIAB]
OR "employee incapacity"[TIAB]
OR "employee incapability"[TIAB])

Limits: Humans, Age>18, Every language, Journal Article, published: 2000-28.03.2017
