# Supplementary material for: The impact of diabetes on labour market participation: a systematic review of results and methods
Source: BMC Public Health. 2019 Jan 7;19:25. doi: 10.1186/s12889-018-6324-6 (PMC6323654; doi:10.1186/s12889-018-6324-6)
Supplement: Supplementary file 2 — Newcastle-Ottawa Scale and quality scores. This file contains a detailed overview and explanation of the Newcastle-Ottawa Scale used to assess quality of the retrieved studies. The file entails also a detailed overview of the scores for each study in each dimension. (PDF 271 kb) [file 12889_2018_6324_MOESM2_ESM.pdf]

The impact of diabetes on labor market participation: a systematic review of results and methods

Sara Pedron, Karl Emmert-Fees, Michael Laxy, Lars Schwettmann*

BMC Public Health

Additional file 2: Newcastle-Ottawa Scale (NOS) and quality scores

*corresponding author

Additional file 2: Newcastle-Ottawa Scale (NOS) and quality scores

Newcastle-Ottawa Scale for cohort studies [16]: adopted scale

Selection

1) Representativeness of the exposed cohort
   a) truly representative of the average member in the community *
   b) somewhat representative of the average member in the community *
   c) selected group of users (e.g. nurses, volunteers)
   d) no description of the derivation of the cohort

2) Selection of the non-exposed cohort
   a) drawn from the same community as the exposed cohort *
   b) drawn from a different source
   c) no description of the derivation of the non-exposed cohort

3) Ascertainment of exposure
   a) secure record (e.g. surgical records) *
   b) structured interview *
   c) written self-report
   d) no description

Comparability

1) Comparability of cohorts on the basis of the design or analysis
   a) study controls for age and socioeconomic features **
   b) study controls for any additional factor (comorbidities and complications)*

Outcome

1) Assessment of outcome
   a) independent blind assessment *
   b) record linkage *
   c) self-report
   d) no description

2) Was follow-up long enough for outcomes to occur (depending on the outcome)?
   a) yes *
   b) no

3) Adequacy of follow-up of cohorts
   a) complete follow-up - all subjects included *
   b) subjects lost to follow-up unlikely to introduce bias - small number of subjects lost (> 60 % follow-up, or description provided of those lost) *
   c) follow-up rate < 60% and no description of those lost
   d) no statement

Modifications:

- For cross-sectional studies, two items were not applicable (Outcome, Items 2 & 3);
- Comparability: according to the original scale, this item could be modified by the authors [16]. We assigned two stars if the study controlled at least for age and socioeconomic variables. We assigned one star if the study did not control for age and socioeconomic variables or if the study additionally controlled for comorbidities and complications without discussing the implications of such modeling choice, since their introduction as confounders could additionally increase bias in the estimation. See the *Discussion* paragraph for a detailed explanation of this point.
- Cross sectional studies could be awarded a maximum of 6 stars;
- Longitudinal (cohort) studies could be awarded a maximum of 8 stars;
- Exception: Yen et al. (2011) were given 1 point for "comparability" although they did not control for age. Since their outcome is precisely "age at retirement" controlling for age would not have been feasible.
- The original scale is actually suitable for evaluating the quality of studies which involve a clinical outcome (i.e. incidence, prevalence, presence of a medical condition) and an exposure of medical or other nature (exposure to different risk factors). Accordingly, the scale assigns one point if the outcome is measured objectively (e.g. through independent blind assessment or record linkage), and no point if the outcome is self-assessed. On the contrary, the measurement of the exposure, following the scale, is awarded one point if it is based on a self-report through a structured interview. In our case, exposure and outcome were reversed: we measured the impact of a medical condition (presence of diabetes) on labor market outcomes. Therefore, the measurement of diabetes presence based on self-reported data through a structured interview was awarded one point. This did not happen, however, for the labor market outcome self-reported information. Since in the majority of studies diabetes status and employment status were measured in the same way, swapping the criteria would not have changed the final result.

Table S.1: Results of quality assessment

	Selection			Comparability	Outcome			
**Study**	Representativeness of the exposed group	Selection of non-exposed group	Ascertainment of exposure	Controls for Confounding	Ascertainment of outcome	Follow-up long enough for outcome to occur	Adequacy of follow up of cohorts	**Quality score**
**Cross-sectional studies**								
Ng et al. (2001) [19]	*	*	*	**				5/6
Bastida et al. (2002) [11]	*	*	*	**				5/6
Yassin et al. (2002) [26]	*	*	*	**				5/6
Vijan et al. (2004) [39]		*	*	**				4/6
Brown et al. (2005) [12]	*	*	*	**				5/6
Klarenbach et al. (2006) [14]	*	*	*	*				4/6
Alavinia et al. (2008) [10]	*	*	*	*				4/6
Harris (2009) [13]	*	*	*	*				4/6
Latif (2009) [15]	*	*	*	**				5/6
Zhang et al. (2009) [28]	*	*	*	*				4/6
Lin (2011) [16]	*	*	*	**				5/6
Minor (2011) [17]	*	*	*	**				5/6
Yen et al. (2011) [27]	*	*		*				3/6
Pit et al. (2013) [21]	*	*		*				3/6
Rumball-Smith et al. (2014) [22]	*	*	*	**				5/6
Smith et al. (2014) [24]	*	*		*				3/6
Seuring et al. (2015) [23]	*	*	*	**				5/6
Minor et al. (2016) [18]	*	*	*	**				5/6
Nielsen et al. (2016) [20]	*		*	**				4/6
Van Der Zee-Neuen et al. (2017) [25]	*	*		**				4/6
**Longitudinal studies**								
Kraut et al. (2001) [33]	*	*	*	**		*		6/8
Vijan et al. (2004) [39]		*	*	**		*	*	6/8
Tunceli et al. (2005) [38]		*	*	**		*	*	6/8
Shultz et al. (2007) [37]		*	*	*		*		4/8
Herquelot et al. (2011) [30]		*		**	*	*	*	6/8
Pit et al. (2012) [36]	*	*				*	*	4/8
Minor (2013) [35]	*	*	*	**		*		6/8
Kang et al. (2015) [31]	*	*	*	*			*	5/8
Majeed et al. (2015) [34]	*	*		*		*		4/8
Ervasti et al. (2016) [29]		*	*	**	*	*	*	7/8
Kouwenhoven-Pasmooij et al. (2016) [32]	*	*	*	*		*	*	6/8

Legenda: * 1 point; ** 2 points.
